# Complement opsonization of HIV-1 results in a different intracellular processing pattern and enhanced MHC class I presentation by dendritic cells

**DOI:** 10.1002/eji.201242935

**Published:** 2013-04-24

**Authors:** Veronica Tjomsland, Rada Ellegård, Adam Burgener, Kenzie Mogk, Karlhans F Che, Garrett Westmacott, Jorma Hinkula, Jeffrey D Lifson, Marie Larsson

**Affiliations:** 1 Division of Molecular Virology, Department of Clinical and Experimental Medicine, Linköping UniversityLinköping, Sweden; 2 Department of Medical Microbiology, University of ManitobaWinnipeg, Canada; 3 National Microbiology LabWinnipeg, Manitoba, Canada; 4 AIDS and Cancer Virus Program, SAIC Frederick, Inc., Frederick National Laboratory for Cancer ResearchFrederick, MD, USA

**Keywords:** Antigen presentation, Antigen processing, Complement opsonization, Dendritic cells, HIV-1

## Abstract

Induction of optimal HIV-1-specific T-cell responses, which can contribute to controlling viral infection in vivo, depends on antigen processing and presentation processes occurring in DCs. Opsonization can influence the routing of antigen processing and pathways used for presentation. We studied antigen proteolysis and the role of endocytic receptors in MHC class I (MHCI) and II (MHCII) presentation of antigens derived from HIV-1 in human monocyte-derived immature DCs (IDCs) and mature DCs, comparing free and complement opsonized HIV-1 particles. Opsonization of virions promoted MHCI presentation by DCs, indicating that complement opsonization routes more virions toward the MHCI presentation pathway. Blockade of macrophage mannose receptor (MMR) and β7-integrin enhanced MHCI and MHCII presentation by IDCs and mature DCs, whereas the block of complement receptor 3 decreased MHCI and MHCII presentation. In addition, we found that IDC and MDC proteolytic activities were modulated by HIV-1 exposure; complement-opsonized HIV-1 induced an increased proteasome activity in IDCs. Taken together, these findings indicate that endocytic receptors such as MMR, complement receptor 3, and β7-integrin can promote or disfavor antigen presentation probably by routing HIV-1 into different endosomal compartments with distinct efficiencies for degradation of viral antigens and MHCI and MHCII presentation, and that HIV-1 affects the antigen-processing machinery.

## Introduction

The most frequent port of entry for HIV-1 is the genital mucosa and in these tissues, DCs can be one of the first cell types that encounter the virus. Mucosal DCs are immature and specialize in sensing danger signals and picking up antigens 1. When activated, DCs migrate to the draining lymph nodes and undertake complex developmental changes, acquiring a mature phenotype and functions optimal for activation of T cells 1. The induction of functional HIV-1 specific CD4^+^ and CD8^+^ T-cell responses can contribute to controlling viral replication and impact disease progression 2–4.

The majority of HIV-1 virions captured by DCs are internalized into endosomal compartments and can either be processed for MHC class II (MHCII) presentation or enter into the cytosol via CD4/coreceptor for MHC class I (MHCI) presentation 5, 6. In late endosomal compartments, virions are processed by proteases and the viral antigens can be loaded onto MHCII molecules, or degraded in the lysosomal compartments 7. Strikingly, a small fraction of endocytosed virions can remain infectious for days in nonacidic intracellular compartments existing in mature DCs (MDCs), but not in immature DCs (IDCs) 8 suggesting differences in trafficking and processing pathways between IDCs and MDCs 8.

HIV-1 binding to CD4/coreceptor leads to presentation of viral antigen by the classical MHCI presentation pathway 5, 6. The fusion of HIV-1 envelope with the cell membrane delivers viral proteins into the cytosol for degradation to peptides by the proteasome. The peptides can then be transported by TAP into ER 5 and subsequently loaded onto MHCI molecules.

Receptor guiding of antigen into specific endosomal compartments is a very important trafficking step that influences antigen presentation 9 as different endosomes exhibit very diverse kinetics for their maturation from early-to-late endosomes and thereby different proteolytic functions and activities 10. An example is the MHCII compartment (MIIC) where the loading of antigenic peptides onto MHCII molecules takes place. An array of endocytic receptors is involved in HIV-1 binding and uptake, among them are CD4, CCR5, CXCR4, integrins, e.g. αMβ2 (CD11b/CD18), and lectins, e.g. DC-SIGN, macrophage mannose receptor (MMR), DEC-205, and DCIR 11–13. Depending on the array of receptors used, the DCs will route antigens to different endosomal compartments with diverse efficiency for processing and presentation 9.

HIV-1 activates the complement system and exists both as free and opsonized particles in vivo 14. HIV-1 escapes complement-mediated virolysis because regulators of the complement system are incorporated in the viral envelope 15. Virions become coated with complement fragments C3b, iC3b, and C3d 15 and can interact with several complement receptors (CR) such as CR1, CR3, and CR4 15. Previous studies have shown that opsonization of HIV-1 can increase infectivity 15 and viral transfer from DCs to target T cells 16. In addition, previous results from our group have shown that opsonized virions are more efficiently internalized by DCs via receptor-mediated endocytosis 12.

The aim of this study was to examine if complement opsonization of HIV-1 affected receptor use, processing, and degradation pathways in IDCs and MDCs leading to MHCI and MHCII antigen presentation and T-cell activation. We found that complement opsonization of HIV-1 led to significantly enhanced MHCI presentation and this implies that a larger amount of the complement opsonized HIV-1 exits the endosomal compartment and gains access to the cytosol and MHCI pathway. Blocking C-type lectins MMR and DEC-205 rerouted HIV-1 to a path leading to higher levels of MHCI presentation and, for MMR, also higher levels of MHCII presentation. Furthermore, blocking β7-integrin enhanced MHCI and MHCII presentation, whereas blockade of CR3 decreased MHCI and MHCII presentation. In addition, expression analysis studies showed that the protease and proteasome activity of IDCs and MDCs was modulated by HIV-1 exposure. These findings indicate that different endocytic receptors guide HIV-1 into endosomal compartments with different properties and efficiencies for degradation of viral antigens and MHCI and MHCII presentation and that HIV-1 affects the antigen-processing machineries.

## Results

### Opsonization of HIV-1 promoted MHC class I (MHCI) presentation by immature DCs (IDCs)

**Figure 1:**
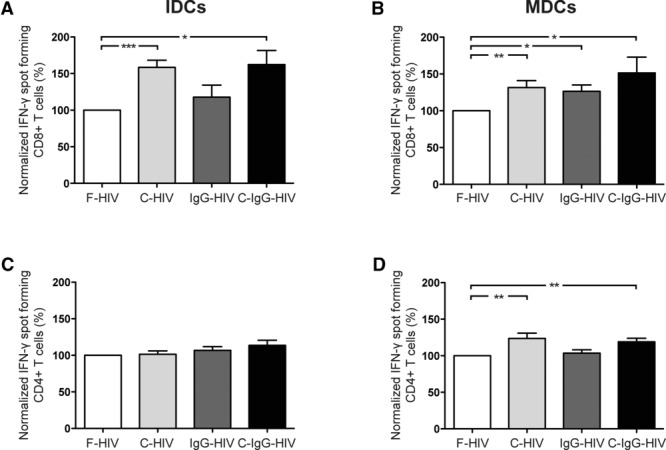
Complement opsonization of HIV-1-enhanced MHCI presentation by IDCs and MDCs. (A–D) IDCs and MDCs (0.15 × 10^6)^ were incubated overnight with mock, free HIV-1_BaL_ (F-HIV), complement opsonized HIV-1_BaL_ (C-HIV), IgG opsonized HIV-1_BaL_ (IgG-HIV), or complement and IgG opsonized HIV-1_BaL_ (C-IgG-HIV) (75 ng p24^CA^ equivalent/group). After the incubation, the different groups of DCs were washed and cocultured with (A and B) an HIV-1 gag p17 SL9 (SLYNTVATL) specific CD8^+^ T-cell clone to assess MHCI presentation or (C, D) HIV-1 p24 LI13 (LNKIVRMYSPTS) specific CD4^+^ T-cell clone to assess MHCII presentation for 12 h. The T-cell activation was assessed by IFN-γ ELISPOT assay. Data are normalized with F-HIV as 100% and shown as mean +SEM of 6–28 independent experiments. *p<0.05, **p<0.005, ***p<0.0001, two-sided paired *t*-test.

We compared how free and opsonized HIV-1 were internalized, processed, and presented on MHCI or MHCII molecules by IDCs and MDCs **(**Fig. 1 A–D: normalized data; Supporting Information Fig. 1A–D: representative experiments). MHCI presentation (CD8^+^ T-cell activation) was significantly enhanced by 63% (*p*<0.0001) for HIV-1 covered with complement fragments (C-HIV) compared with free HIV-1 (F-HIV) (Fig. 1A). In vivo, HIV-1-specific antibodies constitute only a small fraction of all antibodies after seroconversion, so to mimic this we challenged IDCs with HIV-1 opsonized with a combination of immunoglobulin (IgG) (20 μg/mL), neutralizing HIV-1 antibodies (0.2 μg/mL), and complement (C-IgG-HIV). C-IgG-HIV increased MHCI presentation (72%, *p* = 0.012) in IDCs compared to F-HIV (Fig. 1A). However, when HIV-1 was immune complexed only with nonspecific IgG and neutralizing antibodies (IgG-HIV), the level of MHCI presentation by IDCs was similar to that for F-HIV (Fig. 1A). When MDCs were used, MHCI presentation also increased for C-HIV (34%, *p* = 0.002) and C-IgG-HIV (63%, *p* = 0.018) compared with F-HIV (Fig. 1B). The use of IgG-HIV increased MHCI presentation by MDCs with 29% (*p* = 0.02) compared with F-HIV (Fig. 1B).

Surprisingly, no major differences in MHCII presentation were detected between free and the different forms of opsonized virions by IDCs (Fig. 1C). MDCs had a different pattern, with both C-HIV and C-IgG-HIV increasing MHCII presentation (22%, *p* = 0.007: 17%, *p* = 0.003). C-HIV had a similar effect on both MHCI and MHCII presentation for MDC, whereas IgG-HIV only affected MHCI presentation (Fig. 1D). We next assessed the effects F-HIV and C-HIV had on DC expression of costimulatory molecules as they might influence the level of antigen presentation and T-cell activation. We found no significant effect on the expression of CD80, CD86 CD40, or HLADR **(**Supporting Information Fig. 2A–D**)**. We have previously shown the effects of free HIV-1 on the DC ability to activate naïve T cells and found that HIV-1 had negative effects on T-cell proliferation by inducing suppressor T cells with the ability to impair T-cell responses 17, 18. Here we assessed if complement opsonized virions exerted the same effect on DC ability to prime naïve T cells. We found that DCs pulsed with C-HIV had the same negative effect as F-HIV on the induction of T-cell proliferation in naïve bulk T cells **(**Supporting Information Fig. 2E**)**.

### Blockade of HIV-1 usage of CR3 decreased presentat-ion of free and complement opsonized HIV-1 by DCs

**Figure 2:**
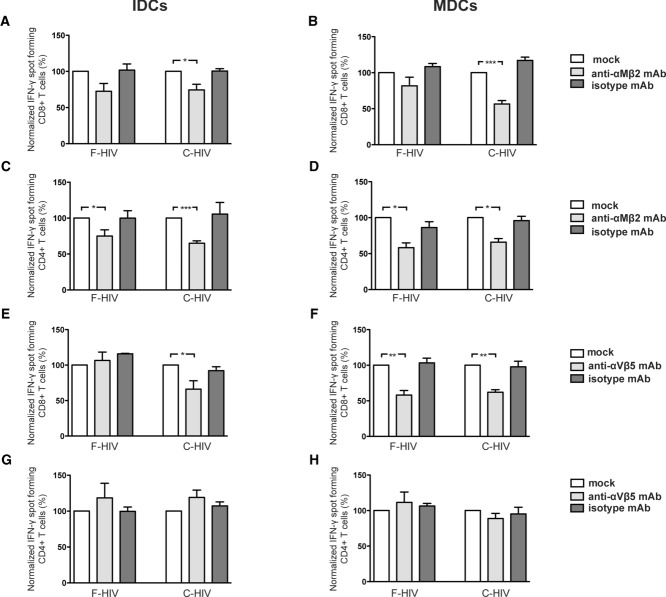
Block of HIV-1 usage of CR3 decreased MHCI and MHCII presentation of free and complement opsonized HIV-1 by DCs. (A–H). IDCs and MDCs were preincubated with (A–D) anti-αMβ2 (CD11b/CD18:CR3) mAb (20 μg/mL), or (E–H) αVβ5 mAb (20 μg/mL) followed by incubation with free HIV-1_BaL_ (F-HIV) or complement opsonized HIV-1_BaL_ (C-HIV). The different groups of DCs were incubated overnight, washed, and cocultured with (A, B, E and F) an HIV-1 gag p17 SL9 (SLYNTVATL) specific CD8^+^ T-cell clone or (C, D, G and H) an HIV-1 p24 LI13 (LNKIVRMYSPTS) specific CD4^+^ T-cell clone. The T-cell responses were assessed by IFN-γ ELISPOT assay. Data are normalized with F-HIV as 100% and shown as mean +SEM of 3 to 6 independent experiments. *p<0.05, **p<0.005, ***p<0.0001, two-sided paired *t*-test.

Integrins are used by many different viruses to attach to and infect host cells 19 and we investigated their role in antigen presentation. IDCs and MDCs were preexposed to blocking antibodies directed against β1 (CD29), αM (CD11b), β2 (CD18), or αVβ5-integrins before challenging the cells with either F-HIV or C-HIV. CR3 (αM/β2) is involved in the enhanced HIV-1 infection of DCs seen for complement opsonized virions 16, 20. Blocking CR3-HIV-1 binding decreased MHCI presentation by IDCs and MDCs of F-HIV (27.4%, *p* = 0.083: 18.2%, *p* = 0.19) and C-HIV (25.5%, *p* = 0.03: 43.5%, *p* = 0.0003) (Fig. 2A and B), indicating that CR3 was responsible for guiding complement-opsonized virions to MHCI presentation. The effect of CR3 blockade was similar for MHCII presentation by IDCs and MDCs with decreased presentation of F-HIV (25%, *p* = 0.045: 48%, *p* = 0.024) and C-HIV (35%, *p* = 0.0005: 34.1%, *p* = 0.022) (Fig. 2C and D). The cell surface receptor αVβ5-integrin is involved in uptake of antigen, e.g. apoptotic cells 21. In addition, αV-integrin has been implicated in HIV-1 infection of macrophages, with reduced HIV-1 replication when this integrin is blocked 22. In IDCs, blocking the αVβ5-integrin did not affect MHCI presentation of F-HIV but reduced MHCI presentation of C-HIV by 33.9% (*p* = 0.04) (Fig. 2E). Using MDCs, MHCI presentation of F-HIV was reduced by 41.9% (*p* = 0.008), and C-HIV by 38%, (*p* = 0.002) (Fig. 2F). Blocking αVβ5-integrin did not affect MHCII presentation for IDCs or MDCs (Fig. 2G and H). This indicates that αVβ5-integrins promoted MHCI presentation of C-HIV for IDCs and of both F-HIV and C-HIV for MDCs.

### Blockade of HIV-1 β7-integrin usage enhanced presentation of free and opsonized HIV-1 by DCs

**Figure 3:**
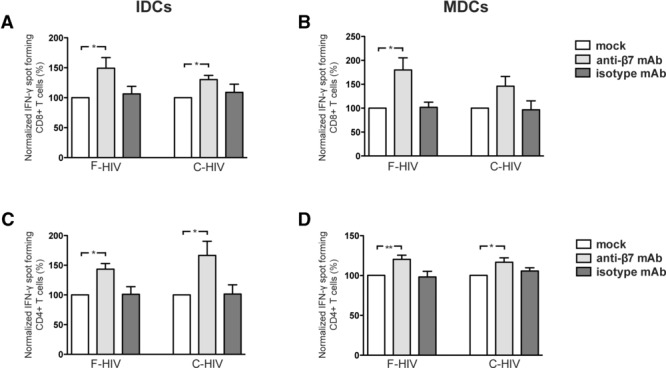
Uptake of HIV-1 involving β7-integrins on DCs significantly decreased both MHCI and MHCII presentation. (A–D) IDCs and MDCs were preincubated with anti-β7 mAb (20 μg/mL) followed by overnight incubation with free HIV-1_BaL_ (F-HIV) or complement opsonized HIV-1_BaL_ (C-HIV). The different groups of DCs were incubated overnight, washed, and cocultured with (A and B) an HIV-1 gag p17 SL9 (SLYNTVATL) specific CD8^+^ T-cell clone or (C and D) an HIV-1 p24 LI13 (LNKIVRMYSPTS) specific CD4^+^ T-cell clone. The T-cell responses were assessed by IFN-γ ELISPOT assay. Data are normalized with F-HIV as 100% and shown as mean +SEM of 4 to 8 independent experiments. *p<0.05, **p<0.005, two-sided paired *t*-test.

On T cells, α4β7-integrins have been implicated in binding HIV-1, targeting virus to susceptible subsets of CD4^+^ T cells and enhancing viral spread 23–25. Blocking the β7-integrins resulted in significantly increased MHCI presentation by IDCs of both F-HIV (49%, *p* = 0.05) and C-HIV (30%, *p* = 0.022). Blockade of β7-integrins on MDCs resulted in increased MHCI presentation using F-HIV (79%, *p* = 0.034) and C-HIV (45% *p* = 0.057) (Fig. 3A and B). MHCII presentation by IDCs was also significantly increased for F-HIV (43%, *p* = 0.02) and C-HIV (66%, *p* = 0.049). In MDCs, blocking β7-integrins gave increased MHCI presentation for both F-HIV and C-HIV (80%, *p* = 0.034: 46% *p* = 0.057) while MHCII presentation increased by 20% (*p* = 0.006) for F-HIV and by 16% (*p* = 0.025) for C-HIV (Fig. 3C and D). These findings indicate that β7-integrin usage by F-HIV and C-HIV disfavored MHCI and MHCII presentation by DCs.

### HIV-1 usage of MMR and DEC-205 routed virions away from MHCI-restricted antigen presentation

**Figure 4:**
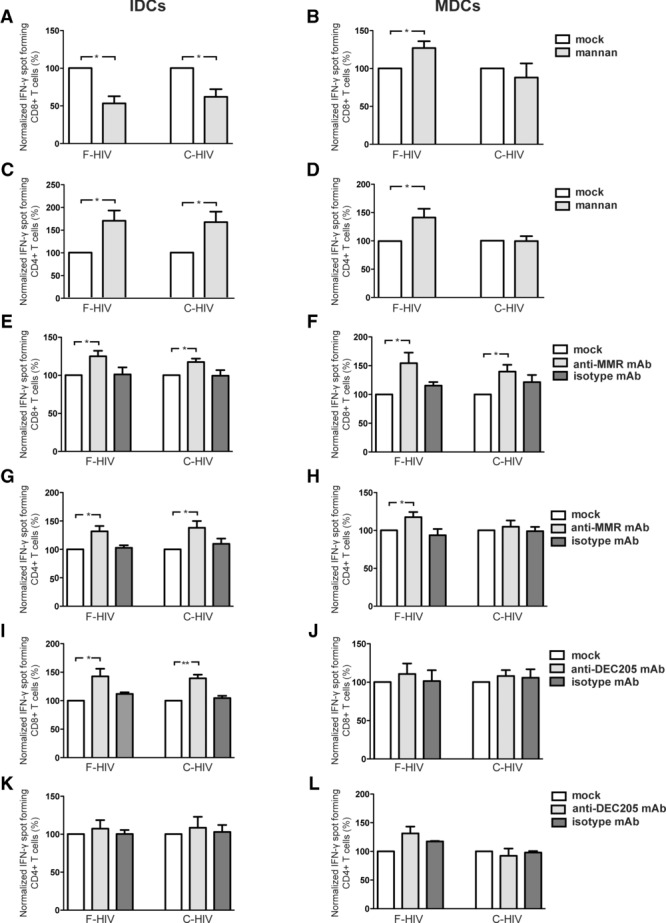
Blocking MMR increased MHCI presentation by IDCs and MDCs. (A–L) IDCs and MDCs were mock preincubated or preincubated with (A–D) mannan (10 mg/mL), (E–H) anti-MMR mAb (20 μg/mL), or (I–L) anti-DEC-205 mAb (20 μg/mL). The different groups of DCs were incubated overnight, washed, and cocultured with an HIV-1 gag p17 SL9 (SLYNTVATL) specific CD8^+^ T-cell clone or an HIV-1 p24 LI13 (LNKIVRMYSPTS) specific CD4^+^ T-cell clone. T-cell responses were assessed by IFN-γ ELISPOT assay. Data are normalized with F-HIV as 100% and shown as mean +SEM of 3 to 6 independent experiments. *p<0.05, **p<0.005, two-sided paired *t*-test.

C-type lectin receptors recognize defined carbohydrates in a Ca^2+^-dependent manner. IDCs were preexposed to mannan, a competitive ligand for C-type lectins, before challenge with F-HIV or C-HIV. Mannan, at a concentration previously shown to block C-type lectins 5, decreased MHCI presentation by IDCs of F-HIV and C-HIV by 47% (*p* = 0.016) and 38% (*p* = 0.02), respectively (Fig. 4A). In MDCs, the blocking of C-type lectins resulted in a moderate but significant increase in MHCI presentation for F-HIV (26%, *p* = 0.045), while no effect was seen for C-HIV (Fig. 4B). In contrast to MHCI, MHCII presentation by IDCs was significantly enhanced for both F-HIV (70%, *p* = 0.018) and C-HIV (67%, *p* = 0.029) when C-type lectins were blocked (Fig. 4C). MDC MHCII presentation was enhanced by 41% (*p* = 0.043) for F-HIV, whereas no effects were seen for C-HIV (Fig. 4D). Taking into consideration our previous findings that mannan decreased the amount of virions internalized in IDCs by 48% for F-HIV and 45% for C-HIV 12, these results indicate that C-type lectins trafficked HIV-1 away from the pathway leading to MHCII presentation and promoted MHCI presentation by IDCs. In MDCs, the C-type lectin composition differs from IDCs and routed F-HIV to an endosomal pathway disfavoring MHCI and MHCII presentation.

Next, we examined the C-type lectin receptors MMR and DEC-205. Blockade of MMR increased presentation via MHCI (24.9%, *p* = 0.027) and MHCII (31.7%, *p* = 0.011) by IDCs (Fig. 4E and G) with similar results for MHCI (54%: *p* = 0.042) and MHCII presentation (17.6%: *p* = 0.036) by MDCs (Fig. 4F and H). Blocking MMR resulted in similar increases in MHCI presentation for C-HIV in IDCs (17.5%: *p* = 0.011) and MDCs (39.8%: *p* = 0.028) (Fig. 4E and F). Furthermore, blocking of MMR on IDCs increased MHCII presentation using C-HIV (38%: *p* = 0.015), whereas this block had no effect for MDCs (Fig. 4G and H). Blockade of DEC-205 enhanced MHCI presentation by IDCs for both F-HIV (42%: *p* = 0.032) and C-HIV (39.3%: *p* = 0.004) (Fig. 4I). For MDCs, there were no effects observed on MHCI presentation for neither F-HIV nor C-HIV (Fig. 4J). MHCII presentation of F-HIV and C-HIV was more or less unaffected by the blocking of DEC-205 for both IDCs and MDCs (Fig. 4K and L). This indicates that HIV-1 binding and internalization via MMR and DEC-205 disfavored MHCI presentation in IDCs, whereas only MMR gave this effect in MDCs.

### Complement opsonized HIV-1 localized in less acid compartments compared to free HIV-1

**Figure 5:**
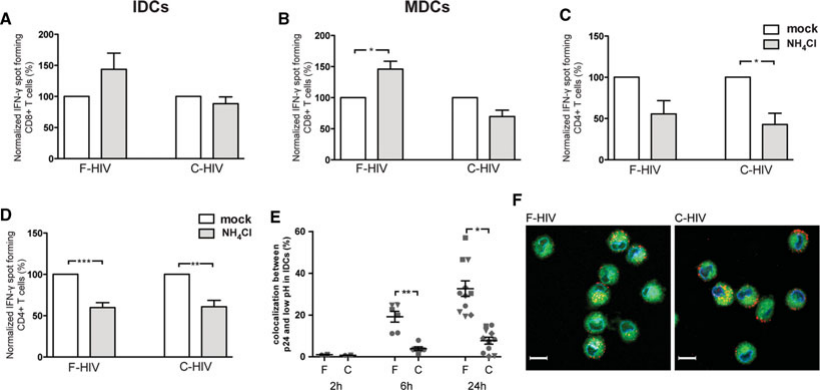
Neutralization of endosomal acidification enhanced MHCI presentation of F-HIV but had no effect for C-HIV. (A–D) IDCs and MDCs were preincubated with mock or NH_4_Cl (60 mM). The different groups of DCs were incubated overnight, washed, and cocultured with (A, B) an HIV-1 gag p17 SL9 (SLYNTVATL) specific CD8^+^ T-cell clone or (C and D) an HIV-1 p24 LI13 (LNKIVRMYSPTS) specific CD4^+^ T-cell clone. T-cell responses were assessed by IFN-γ ELISPOT assay. (E and F) IDCs were exposed to F-HIV or C-HIV and cultured for 2, 6, and 24 h and then stained with a lysosensor that becomes more fluorescent in acidic environments (green), p24 antibody (red), and DAPI (cell nuclei, blue). (E) The colocalization between p24 and acidic compartments was assessed and (F) representative cells are shown. Scale bar, 10 μm. Each symbol represents an individual sample/replicate and data are shown as mean +SEM of 2–12 samples from four independent experiments performed. *p<0.05, **p<0.005, ***p<0.0001, two-sided paired *t*-test.

IDCs and MDCs were pretreated with the weak base NH_4_Cl to neutralize the acidification of their endosomal compartments. This significantly increased MHCI presentation of F-HIV by 43% (*p* = 0.09) in IDCs and by 45% (*p* = 0.036) in MDCs (Fig. 5A and B). Surprisingly, NH_4_Cl had no effect on MHCI presentation of C-HIV (Fig. 5A and B). This difference between free and complement opsonized HIV-1 is not due to changes in the C-HIV use of CD4, coreceptor, and fusion (unpublished observation). In accordance to what we and others have shown previously 5, 26, inhibition of acidification of the endosomal compartment significantly decreased MHCII presentation by IDCs for F-HIV (44.5%, *p* = 0.035) and C-HIV (57.2%, *p* = 0.025). Similar results were also seen using MDCs, with decreased MHCII presentation for F-HIV (40.2%, *p* = 0.0005) and C-HIV (39.2%, *p* = 0.004) (Fig. 5C and D). Neutralization of the acidic endosomal compartments has previously been shown to increase MHCI presentation of free HIV-1 5, 27, whereas the finding that MHCI presentation of C-HIV is unaffected is novel. To investigate if C-HIV was handled and processed differently in the DCs compared to free virions, i.e. ended up in endosomal compartments with slower acidification and/or less acidic environment, we assessed this using a lysosensor that becomes more fluorescent in acidic environments. F-HIV colocalized to a significantly higher degree with acid endosomal compartments than C-HIV at both 6 h (19% versus 4%, *p* = 0.0053) and 24 h (33% versus 7% *p* = 0.0313) (Fig. 5E and F).

### Complement opsonized HIV-1 downregulated prote-ase activity and enhanced proteasome activity in IDCs

**Figure 6:**
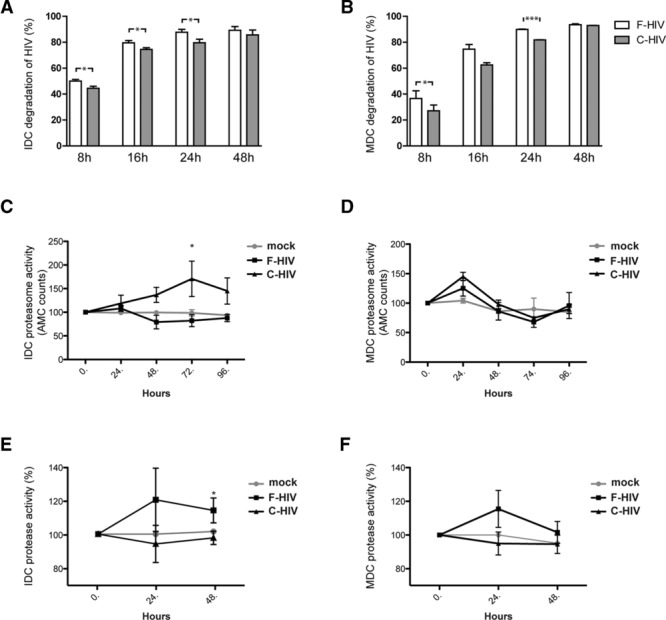
Both F-HIV and C-HIV modulated the proteasome and protease functions in IDCs and MDCs. (A and B) IDCs and MDCs were incubated with free HIV-1_BaL_ (F-HIV) or complement opsonized HIV-1_BaL_ (C-HIV) (75 ng p24^CA^ equivalent/group) for 16, 24, and 48 h. After the incubation, the different groups of DCs were harvested and lysed and the level of HIV-1 p24 assessed by ELISA. Data are normalized with the level of virus present at 2 h set as 100% and are shown as mean ± SEM of 3 to 5 independent experiments performed. (C and D) IDCs and MDCs were incubated with F-HIV or C-HIV (600 ng p24^CA^ equivalent/group) for 0, 24, 48, 72h, and 96 h. After the incubation, the different groups of DCs were harvested and proteasome activity was assessed by measuring the degradation of proteasome substrate III Fluorogenic after 1 h at 37°C. Data are normalized with the level of activity of untreated DCs at 0 h set as 100% and are shown as mean ± SEM of 4 to 7 independent experiments performed. (E and F) IDCs and MDCs were incubated with F-HIV or C-HIV (600 ng p24^CA^ equivalent/group) for 2 h, washed, and cultured for 24 and 48 h. After the incubation, the different groups of DCs were harvested and lysed and protease activity was assessed. Data are normalized with the level of activity of untreated DCs at 0 h set as 100% and are shown as mean ± SEM of 4 to 7 independent experiments performed. *p<0.05, **p<0.005, two-sided paired *t*-test.

Proteolytic degradation of HIV-1 taken up by DCs was slowed when the virions were opsonized by complement, with a lower degradation of antigen derived from C-HIV in IDCs and MDCs at 8, 16, and 24 h compared to F-HIV (Fig. 6A and B). The substrate Suc-LLVY-AMC was used to study proteasome function. Proteasome activity was affected, with increasing activity over time in IDCs exposed to C-HIV, and the highest activity seen at 72 h (70%, *p*<0.05) after exposure (Fig. 6C). The effect of F-HIV was initially slightly enhanced activity followed by decreased function of the proteasome (Fig. 6C). The effect of HIV-1 on MDCs differed from the effect on IDCs as both F-HIV and C-HIV enhanced the activity at 24 h, but decreased the activity at later time points (Fig. 6D). The activity of the proteases was examined and we found that F-HIV enhanced (48 h: 13% (*p*<0.05), while C-HIV decreased protease activity in IDCs and MDCs compared to mock-treated cells (Fig. 6E and F).

### HIV-1 modulated cytosolic factors involved in targeting proteins to the proteasome for degradation

**Figure 7:**
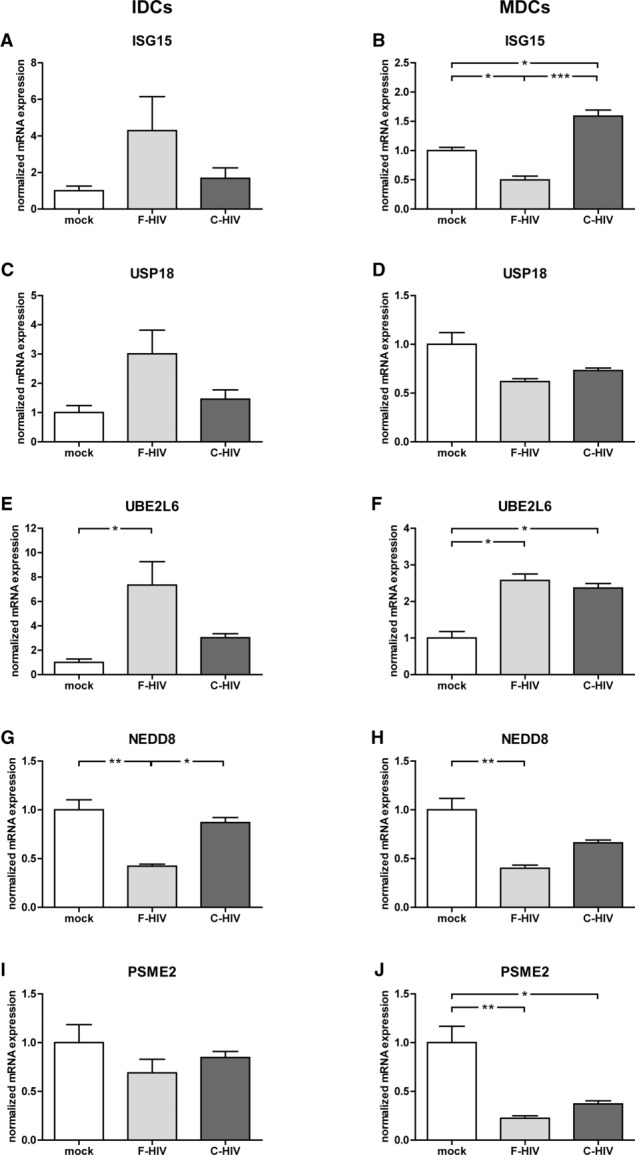
F-HIV and C-HIV modulated factors involved in transport and transport kinetics of protein targeted for proteasome degradation in IDCs and MDCs. (A–J) IDCs and MDCs were incubated with free HIV-1_BaL_ (F-HIV) or complement opsonized HIV-1_BaL_ (C-HIV) (75 ng p24^CA^ equivalent/group) for 48 h. After the incubation, the different groups of DCs were harvested, mRNA was extracted, and gene expression levels of (A and B) ISG15, (C and D) USP18, (E and F) UBE2L6, (G and H) NEDD8, and (I and J) PSME2 were assessed by qRT-PCR. Data are normalized with mock DCs as 100% and are shown as mean +SEM of six independent experiments. *p<0.05, two-sided paired *t*-test.

The mRNA expression levels of an array of proteins, i.e. UBE2L6, ISG15, NEDD8, PSME2, and USP18, involved in the targeting and transportation of proteins for proteasomal degradation were assessed by quantitative RT-PCR (Fig. 7) and/or by quantitative proteomics (Table 1). mRNA expression of components of the IFN-induced ISGylation system (ISG15, USP18, and UBE2L6) was increased by both F-HIV and C-HIV in IDCs at 12 h, with highest impact for the F-HIV-treated cells (Fig. 7A, C, and E). This profile matched the expression of ISG15 pathway proteins (Table 1). The effect of HIV-1 on ISG15 and USP18 mRNA expression in MDCs differed from IDCs, with decreased expression of ISG15 for F-HIV and decreased expression for USB18 for both F-HIV and C-HIV (Fig. 7B, D, and F). We found decreased mRNA expression of components of the NEDDylation system (NEDD8), and proteasome (PSME2) at 12 h for F-HIV and C-HIV in both IDCs and MDCs (Fig. 7G and H) and this correlated to the proteomics data (Table 1).

**Table 1:** Expression of ubiquitin-like pathways in IDCs and MDCs after exposure to F-HIV or C-HIV^a^

iDCs^b^		
Pathway (number of proteins identified)	F-HIV	C-HIV
Ubiquitination, E1 proteins (2)	1.02 (± 0.07)	0.97 (± 0.07)
Ubiquitination, E2 proteins (8)	0.89 (± 0.10)	0.95 (± 0.05)
Ubiquitination, E3 proteins (14)	1.04 (± 0.08)	0.97 (± 0.05)
Deubiquitination (11)	1.03 (± 0.05)	1.01 (± 0.04)
SUMO pathway (7)	1.04 (± 0.05)	0.98 (± 0.04)
NEDD8 pathway (5)	1.76 (± 0.63)	1.33 (± 0.36)
ISG15 pathway (4)	5.54 (± 4.08)	3.38 (± 2.56)

iDCs		
NEDD8 pathway proteins	F-HIV	C-HIV

NEDD8	0.80 (± 0.00)	0.8 (± 0.00)
UBC12 (NEDD8-conjugating enzyme Ubc12)	0.95 (± 0.05)	0.95 (± 0.05)
UBA3 (NEDD8-activating enzyme E1 catalytic subunit)	1.05 (± 0.15)	0.95 (± 0.05)
NAE1 (NEDD8-activating enzyme E1 regulatory subunit)	0.80 (± 0.00)	0.75 (± 0.05)
NUB1 (NEDD8 ultimate buster 1)	3.73 (± 0.53)	2.4 (± 0.46)

iDCs		
ISG15 pathway proteins	F-HIV	C-HIV

ISG15 (ubiquitin-like protein ISG15)	15.87 (± 7.43)	8.9 (± 5.81)
UBA7 (ubiquitin-like modifier-activating enzyme 7)	1.35 (± 0.35)	1.05 (± 0.05)
UBE2L6 (ubiquitin/ISG-conjugating enzyme E2 L6)	2.1 (± 0.50)	1.5 (± 0.37)
TRIM25 (E3 ubiquitin/ISG15 ligase TRIM25)	1.43 (± 0.16)	1.3 (± 0.14)

mDCs^c^		
Pathway (number of proteins identified)	F-HIV	C-HIV

Ubiquitination, E1 proteins (2)	0.99 (± 0.10)	0.96 (± 0.06)
Ubiquitination, E2 proteins (11)	0.89 (± 0.08)	0.89 (± 0.07)
Ubiquitination, E3 proteins (17)	0.90 (± 0.04)	0.94 (± 0.09)
Deubiquitination (19)	0.96 (± 0.04)	0.95 (± 0.04)
SUMO pathway (8)	1.20 (± 0.12)	1.12 (± 0.08)
NEDD8 pathway (5)	1.00 (± 0.05)	1.06 (± 0.07)
ISG15 pathway (4)	0.96 (± 0.07)	0.99 (± 0.07)

a) Expression of proteins in the indicated pathways was determined by quantitative MS proteomics 24 h after exposure of IDCs or MDCs to F-HIV or C-HIV.

b) Data are normalized with mock-treated IDCs as 1 and shown as mean ± SEM of three independent experiments.

c) Data are normalized with mock-treated MDCs as 1 and shown as mean ± SEM of four independent experiments.

## Discussion

The induction of functional HIV-1-specific CD4^+^ and CD8^+^ T-cell responses by DCs can contribute to controlling viremia in infected individuals 2. Consequently, the MHCI and MHCII presentation and priming of naïve CD4^+^ and CD8^+^ T cells can impact the course of infection 3, 4. We have recently shown that complement opsonization of virions enhanced their internalization into DCs 12. Our present study evaluated the impact of opsonization of HIV-1 on processing and antigen presentation. We confirmed that complement opsonization of HIV-1 leads to significantly enhanced MHCI presentation. We show that blocking the C-type lectin receptors MMR and DEC-205 rerouted internalized HIV-1 to a path leading to higher levels of MHCI presentation for IDCs. Furthermore, the blocking of β7-integrin gave enhanced MHCI and MHCII presentation, while inhibition of αMβ2, i.e. CR3, decreased the presentation of HIV-1-derived antigens. We found that free and complement opsonized HIV-1 had diverse influences on the proteolytic activities of proteasome and proteases in DCs. In addition, cytosolic factors involved in the transportation and transport kinetics of proteins targeted for proteasome degradation were impaired in DCs exposed to HIV-1.

Intracellular antigen routing is associated with a number of endocytic receptors including C-type lectins and integrins 10. The initial sorting into different endosomes occurs already at the plasma membrane and is probably dependent on the endocytic receptors and adapter proteins utilized 10 and this distinct guiding of endocytosed antigen is very important for antigen presentation 9. For instance, soluble antigen taken up by MMR is directed to a mildly acidic stable early endosomal compartment for presentation on MHCI molecules, whereas pinocytosed and scavenger receptor endocytosed antigens are targeted more rapidly toward late endosomes where they are processed for MHCII presentation 9. We found that the endocytic receptors used by HIV-1, e.g. lectin receptors and integrins, guided virions into diverse paths leading to different levels of MHCI and MHCII presentation. Wilflingseder et al. 28 have shown that HIV-1 opsonization affects routing into different compartments with a higher amount of IgG and complement IgG opsonized HIV-1 localized in HLA-DR-positive compartments compared with the localization of F-HIV and C-HIV 28.

Integrins have emerged as attachment and/or entry receptors for many viruses, including herpes viruses, rotaviruses, adenoviruses, and HIV-1 19. HIV-1 gp120 can bind and signal through α4β7-integrin in T cells, and it has been proposed that this helps the virus target particularly susceptible CD4^+^ T cells and may play a role in viral spread 25. In addition, αV-integrin has been implicated in HIV-1 infection of macrophages with reduced replication when this integrin is blocked 22. The role of integrins in HIV-1 attachment to DCs and subsequent antigen presentation is not well established. We recently showed that HIV-1 interacts with different integrins expressed on the DCs 12 and blocking use of β7-integrins by HIV resulted in increased MHCI and MHCII presentation by both IDCs and MDCs, indicating that uptake via these integrins disfavors routing of virions for MHCI and MHCII presentation. In addition, the αMβ2/CR3 integrin has been shown by several studies to be involved in the enhanced viral uptake 12 and infection seen for complement opsonized HIV-1 16, 20. We found that CR3 promoted MHCI and MHCII presentation of C-HIV and, to a lesser extent, of F-HIV.

Several C-type lectin receptors have been implicated in antigen capture, uptake, and presentation by DCs 5, 6, 26, 29. MMRs are constitutively internalized and discharge ligands mostly in early endosomes and recycle back to the cell surface, but can also end up in late endosomes depending on the ligand bound 30, 31. DC-SIGN and DEC-205, on the other hand, are internalized upon ligand binding 29. DEC-205 has been shown to be involved in processing and guiding antigen to both MHCI and MHCII presentation by trafficking to late endosomes/MIIC 32, 33. The uptake of HIV-1 via C-type lectins such as DC-SIGN for DCs and MMR by macrophages, is known to enhance trans-infection 34 and this indicates that they route virions to an endosomal compartment with the ability to maintain infectious virus. When DC uptake of HIV-1 involved the C-type lectins MMR and DEC-205, fewer virions were guided to the endosomal MIIC and the cytosol for MHCI presentation. We speculate that these endocytic lectin receptors probably route virions to endosomal compartments less favorable for viral fusion with CD4/coreceptor and formation of MIIC. Another explanation is that endocytic receptors that guide HIV-1 in absence of MMR and DEC-205 are more efficient in guiding to MHCI and MHCII presentation. We have previously shown that a general block of all C-type lectins with mannan decreases uptake of virions by both IDCs and MDCs 12, but even with fewer virions, use of mannan increased MHCII presentation indicating that the exclusion of the array of C-type lectins on DCs rerouted the virus to endosomes efficient in MHCII presentation. β7-integrins and MMR blockage had only small effects on the amount of virions bound and internalized by the DCs 12 but had a bigger impact on the antigen routing into different compartments inside the DCs. This receptor guiding of HIV-1 into different pathways should affect both processing and presentation of viral antigens and infection of the DCs.

A substantial portion of the endocytosed virions accesses the DC cytosol and classical MHCI presentation pathway by binding and fusion via CD4/coreceptor located in the endosomal compartment 5, 35. Most virions that are internalized into the endosomal compartments are proteolysed to different degrees. We found that complement opsonized HIV-1 was degraded slightly slower in DCs. The higher levels of nondegraded C-HIV p24 might be the explanation for the higher infection induced by complement opsonized virions 16, 20 and it might represent viral particles stored in the nonacidic HIV-1-specific compartments 8.

Endosomes mature and gradually become more acidic and this acidification leads to activation of endosomal proteases necessary for MHCII presentation 36. Consequently, preventing acidification gave a decreased MHCII presentation. Neutralization of the acid environment significantly increased the MHCI presentation for F-HIV but had no effect for C-HIV due to the less acid endosomal environment compared to F-HIV. This strongly suggests that the internalization, routing, and processing pathways in DCs differ for F-HIV and C-HIV. Moreover, uptake of C-HIV led to a slower degradation, which gave virions an opportunity to bind to CD4 and CCR5 receptors located in endosomes, fuse, and enter the DC cytosol.

The MHCI antigen-processing steps, i.e. proteasomal degradation, TAP transport, and trimming of peptides, shape the CD8^+^ T-cell responses 4. The proteasome is a cell's major proteolytic machinery and is involved in removal and degradation of misfolded and multi-ubiquitinated proteins 37. In addition, the ubiquitin-proteasome system is the main pathway for degradation of intracellular proteins and is involved in MHCI presentation 5, 38. Inhibition of the protease activity of this structure blocks MHCI processing 38 and presentation 5. HIV-1 proteins have been shown to affect proteasome activity with p24 inducing an altered composition of the immunoproteasome and decreased antigen presentation 39, while Tat has been shown to decrease the activity of the 20S proteasome and slightly increase the activity of the 26S proteasome 40. We found that F-HIV and C-HIV initially enhanced proteasome activity followed by an even more elevated activity for C-HIV but decreased activity for F-HIV in IDCs. This could be a contributing factor for the increased MHCI presentation seen by DCs for C-HIV. Previous studies suggest that a fraction of the HIV-1 particles reaching the cytosol could be degraded by the proteasome in an ubiquitin dependent or independent manner 41, 42. Adaptor proteins, for instance NEDD8 and NEDD8 ultimate buster-1 (NUB1), link the 26S proteasome to ubiquitin-like proteins, such as ISG15 and FAT10, and facilitate proteasomal degradation 37, 43. In addition, ISG15 also acts as a protease inhibitor, which regulates intracellular proteases in APCs 37. We found that both free and C-HIV-affected proteins in the ubiquitin/ubiquitin-like protein pathways, which might affect the DCs’ antigen presentation function.

In conclusion, our study showed clearly that the array of receptors involved in the initial attachment of virions to the DCs influenced the levels of MHCI and MHCII presentation. This occurred by routing of the HIV-1 into endosomal compartments with different properties and efficiencies for degradation, loading of viral antigens on MHCII molecules and for viral fusion and delivery of virus to the cytosol and subsequent MHCI presentation. In addition, HIV-1 modulated the proteolytic systems involved in MHCI and MHCII presentation in DCs.

## Materials and methods

### Propagation of monocyte-derived DCs

Buffy coats were obtained from the Department of Clinical Immunology and Transfusion Medicine, Karolinska University Hospital, Stockholm. Leukapheresis was performed on HLA-A*DRβ1 and HLA-A*0201 positive healthy donors at transfusion medicine, Linköping University Hospital (Ethical permit M173–07). Monocyte-derived DCs were propagated as described previously 5. Maturation was induced day 5 by adding 30 μg/mL poly-I:C (Sigma Aldrich, Stockholm, Sweden) or 30 nM LPS (Sigma Aldrich) for 48 h. On day 7, the immunophenotype of immature and mature DCs was assessed by flow cytometry using FITC- and PE-conjugated mAbs against CD14 and CD83 (BD Biosciences). The purity of the DC cultures was also assessed, and the contamination of other cell types was <1%.

### Virus propagation

HIV-1BaL/SUPT1-CCR5 CL.30 (Lot #P4143: Biological Products Core/AIDS and Cancer Virus Program, SAIC-Frederick, Inc., NCI Frederick) was produced using chronically infected cultures of ACVP/BCP cell line (No. 204), originally derived by infecting SUPT1-CCR5 CL.30 cells (Dr. J. Hoxie, University of Pennsylvania) with an infectious stock of HIV-1BaL (NIH AIDS Research and Reference Reagent Program, Cat. No. 416, Lot No. 59155). Virus was purified and concentrated as previously described 44 and aliquots frozen in liquid N2 vapor. All virus preparations were assayed for infectivity.

### Opsonization of HIV-1

C-HIV was obtained by incubation of HIV-1_BaL_ (30 μg/mL p24 equivalent) with an equal volume of human serum (HS) containing 25% Veronal buffer 12. To obtain C-IgG-HIV, 0.2 μg/mL neutralizing HIV-specific IgG (SMI, Stockholm, Sweden) and 20 μg/mL gamma globulin (Pharmacia, Stockholm, Sweden) were added besides the HS-containing Veronal buffer, whereas IgG-HIV was obtained by adding only the mixture of antibodies. F-HIV was treated with media alone. All groups were incubated for 1 h at 37°C.

### Expansion of HIV-specific CD8^+^ T cells and CD4^+^ T cells

The HLA-A*DRβ1 HIV-1 gag-specific CD4^+^ T-cell clone recognizing the LNKIVRMYSPTSI (LI13) peptide was prepared as described previously 45. The HLA-A*0201 HIV-1 gag p17-specific CD8^+^ T-cell clone recognizing the SLYNTVATL (SL9) peptide (a kind gift from Professor Mario Ostrowski, the University of Toronto, Canada) and the HLA-A*DRβ1 HIV-1 gag-specific CD4^+^ T-cell clone were expanded by coculturing the clones with irradiated feeder cells for 11 days, used directly in assays or cryopreserved until needed.

### ELISPOT assays

IDCs or MDCs were exposed to the following binding and uptake inhibitors for 30 min at 37°C, 60 mM NH_4_Cl, 10 mg/mL mannan; mAbs (20 μg/mL) anti-αM (CD11b) (ICRF44: Ancell Corp. Bayport, USA), anti-β2 (CD18) (TS1/18: BioLegend), anti-β1 (CD29) (JB1A: Millipore), anti-β7 (F1B504: Biosite), anti-αVβ5 (P1F6: Millipore), anti-CD205 (MG38: AbD Serotec), anti-CD206 (15–2: Biosite). In samples where antibodies were used, the matching isotype controls (20 μg/mL) were used. Following incubation with inhibitors DCs (1.5 × 10^5^ DCs/group) were exposed to 75 ng (p24^CA^ equivalent, MOI = 0.15) free HIV-1_BaL_ (F-HIV) or opsonized HIV-1 (C-HIV, IgG-HIV, or C-IgG-HIV) and incubated overnight at 37°C. IDCs and MDCs pulsed with 1 uM LNKIVRMYSPTSI or SLYNTVATL peptides were used as controls for the antigen presentation. DCs were cocultured overnight with HIV-specific CD4^+^ or CD8^+^ T-cell clones in 96-well plates previously coated with 5 μg/mL anti-IFN-γ mAb 1D1-K (Mabtech, Stockholm, Sweden), and the ELISPOT plates were washed and developed, and the detection of spot forming cells was performed as described previously 46.

### LysoSensor assay

IDCs were exposed to F-HIV or C-HIV and cultured for 2, 6, and 24 h. The cells were washed and then incubated with 1 μM LysoSensor Green (Invitrogen, Stockholm, Sweden) for 30 min, stained using p24 (kindly provided by Jorma Hinkula) and Rhodamine Red-X antibodies (Jackson ImmunoResearch, Suffolk, UK), placed on slides using a cytospin and dyed using Vectashield fluorescence medium with DAPI (Vector Laboratories, Peterborough, UK). The cells were scanned using a Zeiss LSM 700 microscope (Carl Zeiss, Stockholm, Sweden) and analyzed using Volocity software (PerkinElmer, MA, USA).

### Degradation assay

MDCs and IDCs (0.15 × 10^6^ DC/well) were challenged with 50 ng (p24^CA^ equivalent/1 × 10^6^ cells/mL: MOI = 0.15) of F-HIV or C-HIV and cultured for 2, 8, 16, 24, and 48 h. All the samples were thoroughly washed to remove unbound virus after 2 h. The samples were lysed in 0.5% Triton X-100 and the amount of HIV-1 was determined by an in-house p24 capture ELISA.

### Proteasome activity assay

IDCs and MDCs (0.2 × 10^6^ DCs/well) were challenged with 3 μg p24 equivalent (MOI = 8.5) per 10^6^ cells of F-HIV, C-HIV, or mock treated. The cells were cultured for 0, 24, 48, 72, or 96 h, harvested, washed, and counted. The pellets were resuspended in reaction buffer (MgCl_2_ (5 mM), ATP (0.5 mM), Triton-X (1%), Glycerol (20%), Tris-HCL (50 mM), and DTT (1 mM) and 0.1 mM proteasome substrate III Fluorogenic (Calbiochem, USA) was added to each sample and incubated for 1 h 37°C. The reactions were quenched by adding 10% SDS (1:10 ratio) and 100 mM Tris Buffer, pH 9.0 (8:1 ratio). The fluorescence of released AMC was recorded at 485 nm excitation and 440 nm emission wavelength using Victor^3^V Multilabel Counter 1420 (PerkinElmer).

### Protease activity assay

The 0.15 × 10^6^ IDCs or MDCs were challenged with F-HIV, C-HIV (3 μg equivalent p24/10^6^ cells, MOI = 8.5) or mock treated and cultured for 0, 24, and 48 h at 37°C. After the incubation, the cells were washed to remove unbound virus. The cells were lysed in 0.5% Triton X-100 and the protease activity in DCs was determined according to the manufacturer's protocol (PF0100, Sigma-Aldrich).

### Quantitative real-time PCR

Total cDNA was produced from DCs exposed for 12 h to mock, F-HIV, or C-HIV using an RNeasy Mini Kit (Qiagen, Sollentuna, Sweden) and a cDNA synthesis kit (Invitrogen). Real-time quantification of transcripts was performed using the SYBR® Green master mix (Applied Biosystems, Stockholm, Sweden) using 5 ng transcript in 5 pM of forward and reverse primers (CyberGene AB, Stockholm, Sweden). β-actin and GADPH served as housekeeping genes (See Supporting Information Table 1 for primer sequences).

### Preparation of IDCs for MS/MS proteomics analysis

IDCs (1 × 10^6^ cells/mL) were challenged with mock, F-HIV, C-HIV, or C-IgG-HIV (3 μg equivalent p24/10^6^ cells, (MOI = 8.5) and cultured for 24 h. The cells were washed and lysed in SDS lysis buffer. The samples were incubated for 5 min at 95°C, sonicated, and 100 μg of the cell lysates were digested with trypsin into peptides and labeled with iTRAQ according to manufacturer's protocol (AB Sciex, Foster City, CA, USA). Next, the peptides were fractionated by RP-LC and analyzed by an LC-MS/MS using an Easy nLC coupled to an LTQ Orbitrap Velos MS (Thermo Scientific, West Palm Beach, FL, USA). Two independent technical replicates were performed for each sample.

### Statistical analysis

The statistical program GraphPad Prism 5 (GraphPad Software, La Jolla, CA, USA) was used for analysis of all data and a two-sided paired *t*-test was used to test for statistical significance. Results were considered statistically significant if *p* ≤ 0.05. All experiments were performed in triplicates with the exception of MS proteomics that was performed in duplicates. When experimental values were normalized, F-HIV or mock was set to 100% or 1. RT-qPCR results were normalized for variation between plates as described by Rieu et al 47. All values ± SEM.

## Abbreviations

CR3: complement receptor 3
IDC: immature DC
MDC: mature DC
MHCI: MHC class I
MHCII: MHC class II
MMR: macrophage mannose receptor

## Supplementary material

As a service to our authors and readers, this journal provides supporting information supplied by the authors. Such materials are peer reviewed and may be re-organized for online delivery, but are not copy-edited or typeset. Technical support issues arising from supporting information (other than missing files) should be addressed to the authors.

Table S1. Primer sequences.

**Figure S1: *Complement opsonization of HIV-1 enhanced MHCI presentation by IDCs and MDCs* (A-D)** IDCs and MDCs (0.15 x106) were incubated over night with mock, free HIV-1BaL (F-HIV), complement opsonized HIV-1BaL (C-HIV), IgG opsonized HIV-1BaL (IgG-HIV), or complement and IgG opsonized HIV-1BaL (C-IgG-HIV) (75ng p24CA equivalent/group). After the incubation the different groups of DCs were washed and cocultured with a HIV-1 gag p17 SL9 (SLYNTVATL) specific CD8^+^ T-cell clone to assess MHCI presentation **(A-B)** or HIV-1 p24 LI13 (LNKIVRMYSPTS) specific CD4^+^ T-cell clone to assess MHCII presentation **(C-D)** for 12h. The T-cell activation was assessed by IFN-g ELISPOT assay and the amount of spot forming cells (SFC) was measured. Data are shown as mean ±SEM and one representative experiment, with triplicate values, out of 6-28 experiments performed.

**Figure S2: *Complement opsonization of HIV did not affect DC expression of costimulatory molecules and their ability to prime naïve T cell responses*. (A-D)** DCs were exposed to mock, F-HIV or C-HIV for 48h and the level of expression of CD40, CD80, CD86, and HLA DR was assessed by staining with PE-conjugated antibodies and analyzing by flow cytometry. Poly I:C (25 ug/mL) was used as a positive control for maturation. Data are shown as mean ±SEM of three experiments performed **(E)** DC were pulsed with mock, F-HIV or C-HIV overnight, washed twice, and cocultured with naïve bulk T cells at a ratio of 1:10. Priming cultures were restimulated with 10 000 DC/well after 7 days of coculture and T-cell proliferation measured on day 8 by 3H-Thymidine incorporation. Data are shown as mean ±SEM of six experiments performed.
