# Supplementary material for: Complement opsonization of HIV-1 results in a different intracellular processing pattern and enhanced MHC class I presentation by dendritic cells
Source: Eur J Immunol. 2013 Apr 24;43(6):1470–83. doi: 10.1002/eji.201242935 (PMC3738931; doi:10.1002/eji.201242935)
Supplement: Supplementary file 1 [file eji0043-1470-SD1.pdf]

European Journal of Immunology

Supporting Information

for

DOI 10.1002/eji.201242935

Veronica Tjomsland, Rada Ellegård, Adam Burgener, Kenzie Mogk, Karlhans F. Che, Garrett Westmacott, Jorma Hinkula, Jeffrey D. Lifson and Marie Larsson

Complement opsonization of HIV-1 results in a different intracellular processing pattern and enhanced MHC class I presentation by dendritic cells

© 2013 WILEY-VCH Verlag GmbH & Co. KGaA, Weinheim

Supplementary data

Supplementary Table 1. Primer sequences.

**UBE2L6**	**F**	5'-ATG GCG AGC ATG CGA GTG GTG-3'
	**R**	5'-GGT CAG CGA GGT CCA TCC GC-3'
**ISG15**	**F**	5'-TGG TGG ACA AAT GCG ACG AA-3'
	**R**	5'-CAG GCG CAG ATT CAT GAA C-3'
**NEDD8**	**F**	5'-CAA GAG TGC TGG AGG GCG GC-3'
	**R**	5'-ACC ACC TCC TCC TCT CAG AGC CA-3'
**PSME2**	**F**	5'-CGA AAG CCC GGG CGA CTA GC-3'
	**R**	5'-GAT GTC CAG TGG GGC CCG GA-3'
**USP18**	**F**	5'-ACA GAC CTG CTG CCT TAA CTC CTT-3'
	**R**	5'-TGG GCA CCG TGA TCC TCT TCA ATA-3'
**GADPH**	**F**	5'- CCA CCA TGG AGA AGG CTG GGG CTC-3'
	**R**	5'- AGT GAT GGC ATG GAC TGT GGT CAT-3'
**β-actin**	**F**	5'- GCT CGT CGT CGA CAA CGG-3'
	**R**	5'- CAA ACA TGA TCT GGG TGA TCT TCT C-3'

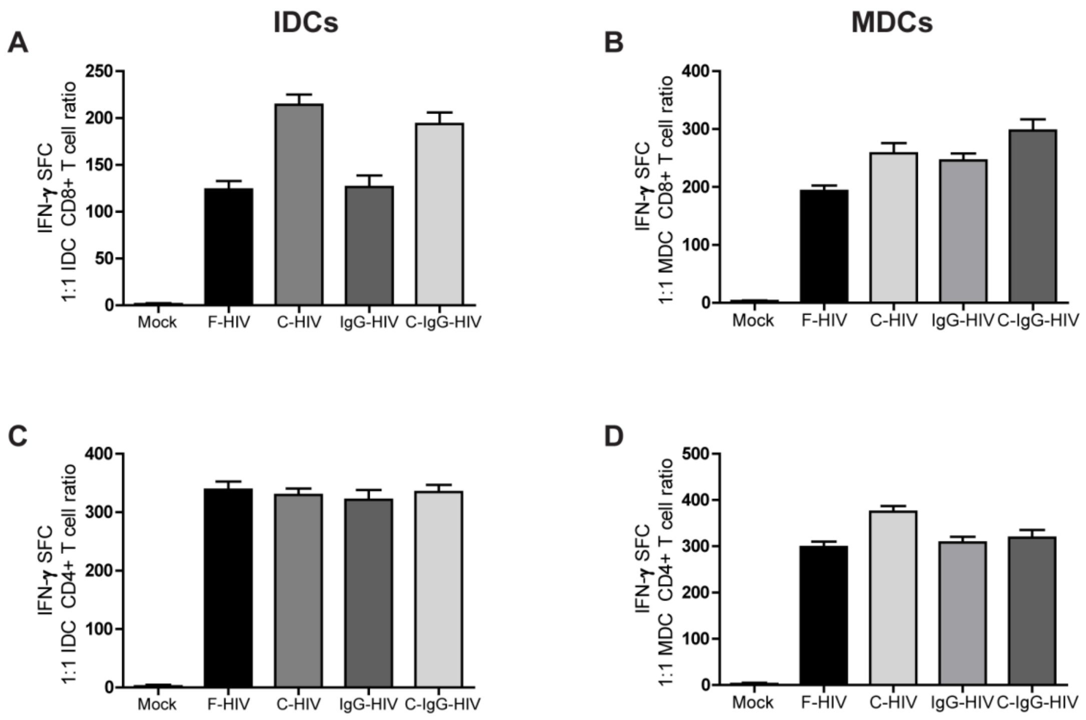

Supplementary Figure 1: ***Complement opsonization of HIV-1 enhanced MHCI presentation by IDCs and MDCs*** **(A-D)** IDCs and MDCs (0.15 x$10^{6)}$ were incubated over night with mock, free HIV-1_{BaL} (F-HIV), complement opsonized HIV-1_{BaL} (C-HIV), IgG opsonized HIV-1_{BaL} (IgG-HIV), or complement and IgG opsonized HIV-1_{BaL} (C-IgG-HIV) (75ng $p24^{CA}$ equivalent/group). After the incubation the different groups of DCs were washed and cocultured with a HIV-1 gag p17 SL9 (SLYNTVATL) specific $CD8^+$ T-cell clone to assess MHCI presentation **(A-B)** or HIV-1 p24 LI13 (LNKIVRMYSPTS) specific $CD4^+$ T-cell clone to assess MHCII presentation **(C-D)** for 12h. The T-cell activation was assessed by IFN-γ ELISPOT assay and the amount of spot forming cells (SFC) was measured. Data are shown as mean ±SEM and one representative experiment, with triplicate values, out of 6-28 experiments performed.

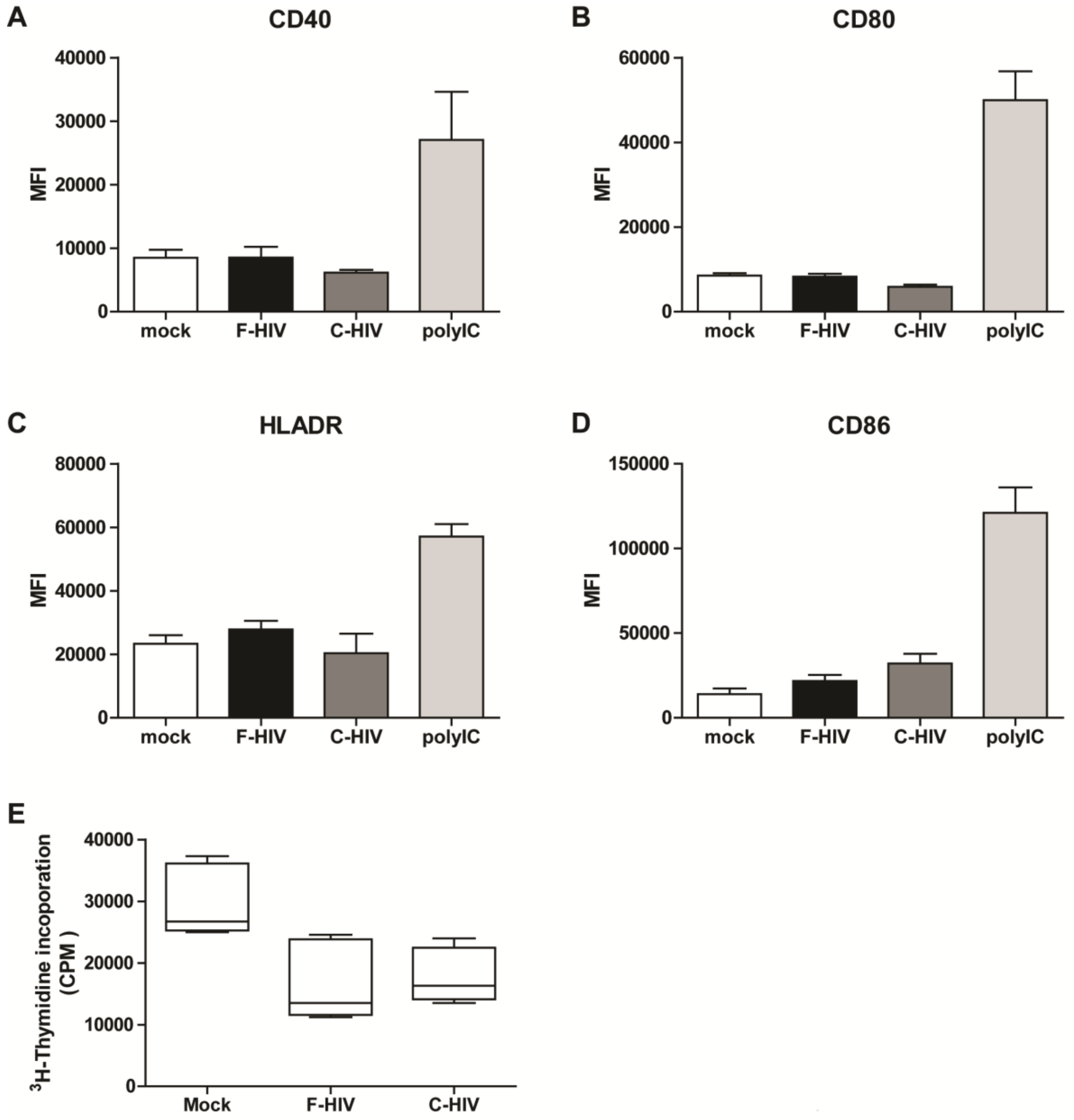

Supplementary Figure 2: ***Complement opsonization of HIV did not affect DC expression of costimulatory molecules and their ability to prime naïve T cell responses.*** **(A-D)** DCs were exposed to mock, F-HIV or C-HIV for 48h and the level of expression of CD40, CD80, CD86, and HLA DR was assessed by staining with PE-conjugated antibodies and analyzing by flow cytometry. Poly I:C (25ug/ml) was used as a positive control for maturation. Data are shown as mean ±SEM of 3 experiments performed **(E)** DC were pulsed with mock, F-HIV or C-HIV overnight, washed twice, and cocultured with naïve bulk T cells at a ratio of 1:10. Priming cultures were restimulated with 10 000 DC/well after 7 days of coculture and T-cell proliferation measured on day 8 by 3H-Thymidine incorporation. Data are shown as mean ±SEM of 6 experiments performed.

Supplementary Method

Allogeneic DC–T cell proliferation assay

Naive $CD4^+$ and $CD8^+$ T cells were negatively selected from PBMCs using magnetic beads (Miltenyi Biotec Auburn, CA, USA) by depleting monocytes (CD14), B cells (CD19), NK cell, (CD56), and memory T cells (CD45RO). Mock, F-HIV, or C-HIV exposed MDCs were harvested, washed twice, and cocultured in 5% PHS with CFSE-labeled (Fisher Scientific) naive bulk T cells at a ratio of 1:10 in 96-well plates. Assays were restimulated after 7 days and T cells were analyzed 1 day after restimulation. Mock DC-T-cell cocultures were used as standard to evaluate the effects HIV-1 had on T-cell priming. T-cell proliferation was assessed at several time points between day 3 and 11 by 4 mCi of 3H-Thymidine incorporation (Amersham Pharmacia).
